# Artificial intelligence model system for bone age assessment of preschool children

**DOI:** 10.1038/s41390-024-03282-5

**Published:** 2024-05-27

**Authors:** Chengcheng Gao, Chunfeng Hu, Qi Qian, Yangsheng Li, Xiaowei Xing, Ping Gong, Min Lin, Zhongxiang Ding

**Affiliations:** 1 Department of Radiology, Hangzhou First People’s Hospital, Hangzhou, China; 2 The Fourth School of Clinical Medicine, Zhejiang Chinese Medicine University, Hangzhou, China; 3 Department of Radiology, The Third Affiliated Hospital of Zhejiang Chinese Medicine University, Hangzhou, China; 4 Rehabilitation Medicine Center, Department of Radiology, Zhejiang Provincial People’s Hospital, Affiliated People’s Hospital, Hangzhou Medical College, Hangzhou, China; 5 Deepwise AI Lab, Beijing, China; 6 College of Humanities and Management, Zhejiang Chinese Medical University, Hangzhou, China; 7 Key Laboratory of Clinical Cancer Pharmacology and Toxicology Research of Zhejiang Province, Hangzhou, China

## Abstract

**Backgroud:**

Our study aimed to assess the impact of inter- and intra-observer variations when utilizing an artificial intelligence (AI) system for bone age assessment (BAA) of preschool children.

**Methods:**

A retrospective study was conducted involving a total sample of 53 female individuals and 41 male individuals aged 3–6 years in China. Radiographs were assessed by four mid-level radiology reviewers using the TW3 and RUS–CHN methods. Bone age (BA) was analyzed in two separate situations, with/without the assistance of AI. Following a 4-week wash-out period, radiographs were reevaluated in the same manner. Accuracy metrics, the correlation coefficient (ICC)and Bland-Altman plots were employed.

**Results:**

The accuracy of BAA by the reviewers was significantly improved with AI. The results of RMSE and MAE decreased in both methods (*p* < 0.001). When comparing inter-observer agreement in both methods and intra-observer reproducibility in two interpretations, the ICC results were improved with AI. The ICC values increased in both two interpretations for both methods and exceeded 0.99 with AI.

**Conclusion:**

In the assessment of BA for preschool children, AI was found to be capable of reducing inter-observer variability and enhancing intra-observer reproducibility, which can be considered an important tool for clinical work by radiologists.

**Impact:**

- The RUS-CHN method is a special bone age method devised to be suitable for Chinese children.
- The preschool stage is a critical phase for children, marked by a high degree of variability that renders BA prediction challenging.
- The accuracy of BAA by the reviewers can be significantly improved with the aid of an AI model system.
- This study is the first to assess the impact of inter- and intra-observer variations when utilizing an AI model system for BAA of preschool children using both the TW3 and RUS-CHN methods.

## Introduction

Bone age (BA), an indicator of biological age,^1–3^ is determined through the assessment of hand-wrist X-rays to gauge skeletal maturity. This assessment serves as a reflection of the actual growth and development in children. The disparity between skeletal age and chronological age (CA) is pivotal in bone age assessment (BAA) for monitoring growth irregularities in children, verifying endocrine-related diagnoses, forecasting adult height, and appraising treatment efficacy.^1^ In China, two noteworthy BAA methods are employed, specifically the Tanner–Whitehouse III (TW3) method.^4^ and the China 05 RUS–CHN (RUS–CHN) method.^5^ Presently, the TW3 method stands as the globally prevalent BAA method.^6^ This scoring system having undergone two modifications, evaluates the skeletal maturity of individuals.^4^ The RUS-CHN method, introduced by Chinese researchers in 2005, was devised to be more suitable for Chinese children compared to the globally adopted TW3 method.^5^ Consequently, the development of a high-performance automatic assessment system that combines these two BA evaluation methods holds substantial clinical significance.

Computer hardware capabilities advance and software technology continually improves, the integration of artificial intelligence (AI) technology in the medical domain has grown increasingly commonplace.^7^ Over recent years, deep learning (DL) models founded on extensive data have played pivotal roles in various aspects of disease diagnosis.^8,9^ Given the relatively straightforward nature of BAA, which entails the assessment of a single wrist X-ray image utilizing a standardized scoring system, it represents an ideal avenue for training DL solutions and crafting AI model systems. Multiple AI systems related to BA have been developed globally, including BoneXpert, GoogLeNet, and OxfordNet. These systems yield results comparable to traditional manual BAA, offering the merits of objectivity, efficiency, and time-saving.^8–11^ Numerous studies have demonstrated that AI application in BAA enhances the diagnostic accuracy of radiologists while reducing evaluation time.^10,12,13^ Some of these studies have concentrated on the assessment consistency among radiologist observers although only a limited number have delved into intra-observer variability. The preschool stage is a critical phase for monitoring the growth and development of children in China,^14^ marked by a high degree of variability that renders BA prediction challenging. Insufficient studies employed the TW3 and RUS-CHN methods for assessing the BA of preschool children.

Hence, the selection of an AI model system capable of assisting radiologists in BAA is crucial. The DL software we employ can accommodate a range of BAA methods, encompassing both the TW3 and RUS-CHN methods, for appraising the hand-wrist of BA in preschool children. This study’s objective is to investigate the impact of AI model software on inter-observer consistency and intra-observer reproducibility in X-ray BAA for preschool children.

## Materials and methods

### Data acquisition

**Fig. 1:**
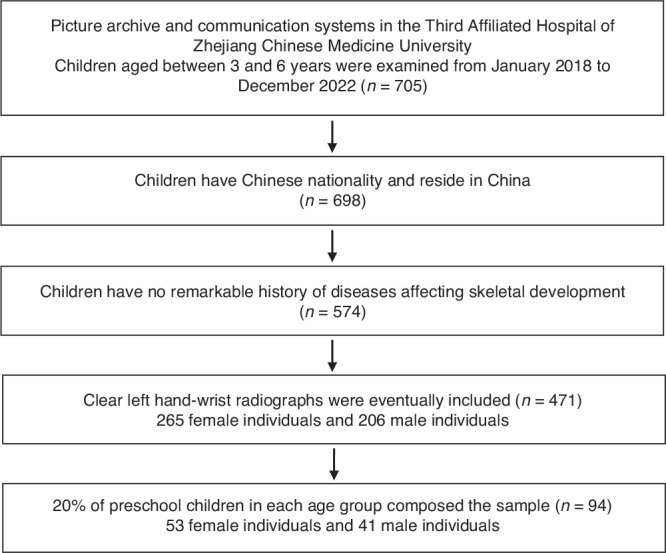
Flowchart of the sample selection process. A total of 53 female individuals and 41 male individuals were selected in this retrospective study.

This retrospective study involved the selection of 471 left hand-wrist radiographs sourced from the Third Affiliated Hospital of Zhejiang Chinese Medicine University in Hangzhou, China. The radiographs were all retrieved from the Picture Archive and Communication Systems (PACS) spanning the period between January 2018 and December 2022. The inclusion criteria stipulated: 1) children aged between 3 and 6 years, 2) children of Chinese nationality and residing in China, and 3) children with no significant medical history of conditions affecting skeletal development. Unclear left hand-wrist radiographs were excluded from the study. We performed stratified random sampling among preschool children aged 3–6, with 20% of the data from each age group included in the study sample. This resulted in a total of 53 female individuals and 41 male individuals (Fig.1, Table 1). None of the cases in the study were involved in the training and validation of the AI model system.

**Table 1:** Demographic characteristics of the 471 Chinese children.

Sex	Female				Male				Total
Age group	3	4	5	6	3	4	5	6	/
Number	53	67	68	77	45	47	53	61	471
Selected	11	13	14	15	9	9	11	12	94
%	11.7	13.8	14.9	15.9	9.6	9.6	11.7	12.8	100

### Imaging examination method

All medical images were captured using the Canon CMP200 scanner from Canon Medical Systems scanner. Radiographs of the left hand-wrist were acquired using the following exposure settings: 1) Tube voltage at 75 kV, tube current at 200 uA, film distance approximately 90 cm, and exposure time of 500 ms. 2) The subject’s left palm was positioned at the center of the irradiation field, pressed firmly against the detection plate, fingers naturally extended, and the centerline aligned with the base of the third metacarpal bone. 3) The displayed area encompassed all carpal, metacarpal, and phalangeal bones, in addition to the distal radius and ulna, covering a range of 3–4 cm. 4) Radiation protection measures were taken to safeguard other body parts.

### Bone age assessment

#### Radiograph evaluation

The BA films were stored in Digital Imaging and Communications in Medicine format within the PACS, with the subject’s information anonymized. All readers were kept unaware of the clinical medical history and patient characteristics. Each radiograph for BAA was assessed using two methods: 1) the TW3 method, which scores the skeletal maturity of each hand and wrist bone, as published in 2001,^4^ and 2) the RUS–CHN method, which analyzes the skeletal development standards of the hand and wrist for Chinese children, version 05-I, as published in 2006.^5^

#### Reference bone age

Two associate chief radiology physicians, trained in the BAA system, were recruited for this study. Each of them possessed over 15 years of experience in BAA and evaluated more than 2000 films annually. They conducted BAA for the hand-wrist radiographs in the samples, following a double-blind approach, and obtained BA values using both the TW3 method and RUS-CHN method. The average of their results was calculated to establish the reference BA standard for this study.

#### AI model development for BAA

The AI model system named Dr. Wise for BAA in this study was developed by the DeepWise Inc. The software had received clinical approval from China’s National Medical Products Administration for Class III medical device certification in 2022 and gained widespread acceptance for clinical use. Hand and wrist landmark detection was automatically performed by this software to identify region of interests for epiphyseal development ratings. Users could overwrite the initial BAA proposed by AI models. Previous study disclosed that the AI model was trained using more than ten thousand hand radiographs from six centers in China.^15^ The mean absolute error (MAE) between the Dr. Wise AI model’s results and the reference standard was 0.266 and 0.249 years for the TW3 method and the RUS-CHN method, respectively.

### Radiograph interpretation

A cross-study design was adopted for radiograph interpretation. Four mid-level radiologists, certified by the committee and possessing 3–5 years of experience, were selected as reviewers. They underwent training on BAA systems before conducting assessments on the samples, familiarizing themselves with the reading and reporting protocols. Following a double-blind approach to image evaluation, they performed BAA using both the TW3 method and the RUS–CHN method.

**Fig. 2:**
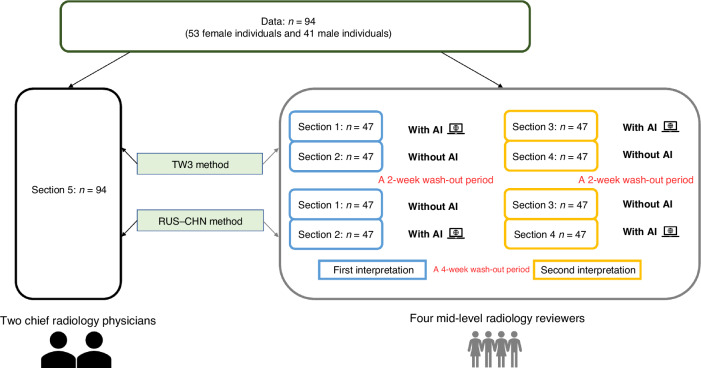
Flowchart of image interpretation. Flowchart of image interpretation by two physicians and four reviewers. (Note: The database was randomly and equally divided into Section 1 & 2 for the 1st interpretation and Section 3 & 4 for the 2nd interpretation. Section 1~4 were not the same for each reviewer).

All researchers carried out two radiograph interpretations, with a 4-week wash-out period separating the two interpretations. To minimize memory-related errors, a two-step random cross-reading method was employed for each interpretation. The sample database’s radiographs were randomly divided into two portions: one for interpretation with AI assistance and the other for interpretation without AI. Radiographs were independently evaluated by reviewers under different conditions. Reviewers were informed of the sex and chronological age of subjects, but were blinded of each other’s BAA results, as in the case of their daily clinical practices. Bone age assessment was conducted by reviewers relying on their own experience, and results were directly derived and recorded. While in the interpretation with AI, the reviewers assessed bone age through the following 3 steps: 1) Reviewers were asked to undergo an independent process of BAA. 2) The BAA results generated by the AI model system were then sent to the reviewers’ computers. 3) Reviewers were guided to make comparations and corrections of the previous independent results, and complete the final BAA report with the assistance of AI. A 2-week wash-out period was maintained between the two steps. The crossover study design is depicted in Figs. 1 and 2.

### Data analysis

Data analysis was conducted using SPSS statistical software (version 26.0, SPSS, Inc, Chicago, IL). With the reference standard’s average value as the basis for comparison, the root mean square error (RMSE), MAE, and the percentage of errors within 0.50 years and 0.25 years for the first BAA interpretation were calculated and compared among the four mid-level radiologists under the conditions of “no AI” and “with AI,” respectively. Quantitative data were examined for normality using histograms. Paired t-tests were used to compare the MAEs of reviewers with and without AI, with significance set at a *p*-value < 0.001. ICCs, Bland-Altman plots with the mean difference and 95% limits of agreement (LoA) results were generated to compare the results with and without AI for the four reviewers (Reviewers 1–4) in the 1st and 2nd interpretations. Intra-observer reproducibility among reviewers was assessed using the ICCs and 95% LoA results, comparing BAA results of the same reviewer in two different interpretations.

## Results

### Accuracy of AI model system in BAA

**Fig. 3:**
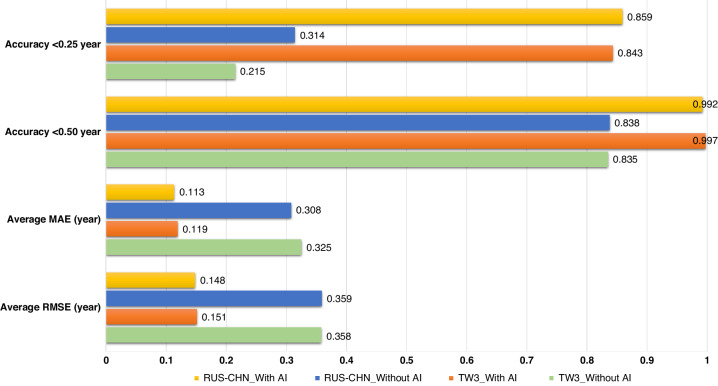
Accuracy metrics. RMSE, MAE value and interpretation accuracy of the TW3 and RUS-CHN methods with and without AI in the 1st interpretation.

The results of BAA using the TW3 method and RUS-CHN methods, both with and without AI assistance, were compared with the reference standard, as shown in Fig. 3. In the first interpretation, the accuracy of the TW3 method’s results for BAA improved significantly with AI assistance compared to without AI. RMSE decreased from 0.358 to 0.151 and MAE reduced from 0.325 to 0.119 (*p* < 0.001). The accuracy within 0.50 years increased from 83.5% to 99.7%, and the accuracy within 0.25 years rose significantly from 21.5% to 84.3%. Similarly, when utilizing the RUS-CHN method for BAA, the results’ accuracy was enhanced with AI assistance compared to without AI. RMSE decreased from 0.359 to 0.148 and MAE decreased from 0.309 to 0.113 (*p* < 0.001). The accuracy within 0.50 years increased from 83.8% to 99.2%, and the accuracy within 0.25 years increased significantly from 31.4% to 85.9%.

### Comparison of inter-observer agreement

**Fig. 4:**
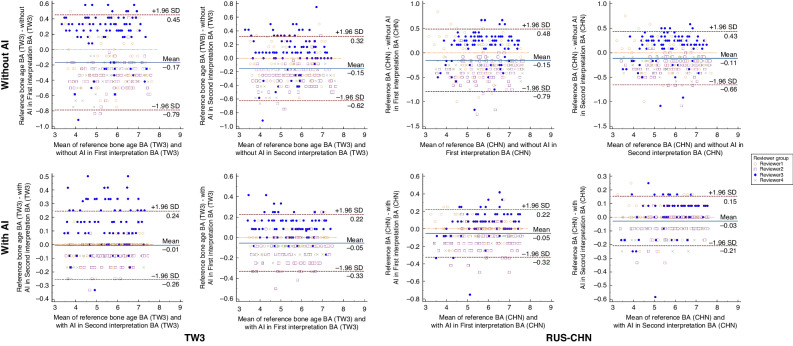
Inter-observer variability. The Bland-Altman plots showing the differences between the reviewers’ BA and the reference standard BA with (bottom) and without AI (top) in both TW3 and RUS-CHN methods.

In the inter-observer agreement comparison for the TW3 method, the ICC values, mean differences and 95% LoA results of the four reviewers improved when using the AI model system. Their ICC values increased from 0.956 to 0.991 in the first interpretation and from 0.974 to 0.993 in the second interpretation. Their mean difference and 95% LoA results increased from −0.17 (95% LoA: −0.79, 0.45) to −0.01 (95% LoA: −0.26, 0.24) in the first interpretation and from −0.15 (95% LoA: −0.62, 0.32) to −0.05 (95% LoA: −0.33, 0.22) in the second interpretation. In the case of the RUS-CHN method, the ICC values, the mean difference and 95% LoA results also saw improvements. Their ICC values increased from 0.950 to 0.991 in the first interpretation and from 0.963 to 0.996 in the second interpretation with AI assistance. Their mean difference and 95% LoA results increased from −0.15 (95% LoA: −0.79, 0.48) to −0.05 (95% LoA: −0.32, 0.22) in the first interpretation and from −0.11 (95% LoA: −0.66, 0.43) to −0.03 (95% LoA: −0.21, 0.15) in the second interpretation with AI assistance (Fig. 4).

### Comparison of intra-observer reproducibility

**Fig. 5:**
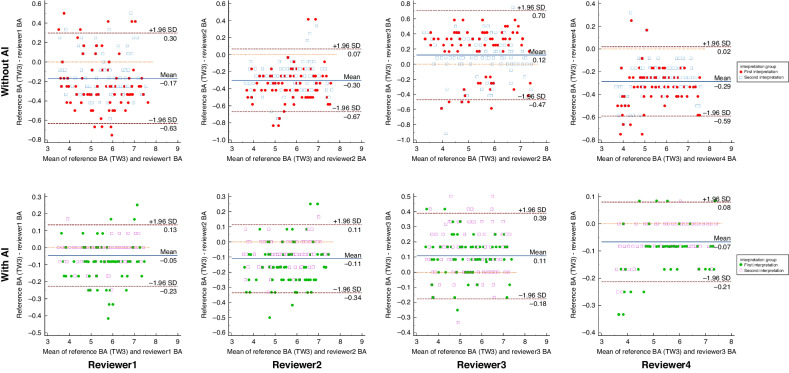
Intra-observer reproducibility (TW3 method). The Bland–Altman plots showing the intra-observer reproducibility among reviewers using TW3 method with (bottom) and without AI (top).

**Fig. 6:**
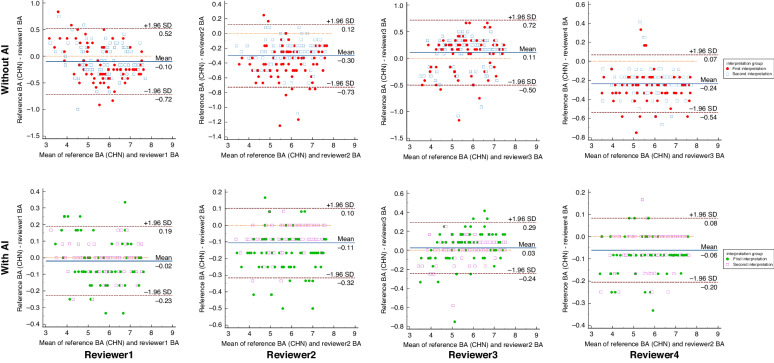
Intra-observer reproducibility (RUS-CHN method). Bland-Altman plots showing the intra-observer reproducibility among reviewers using RUS-CHN method with (bottom) and without AI (top).

In the two interpretations, the intra-observer reproducibility of the TW3 method showed that the ICC and 95% LoA results for each reviewer with the assistance of the AI model system were slightly improved than without AI. Similar results were observed when using the RUS-CHN method for BAA. Furthermore, with AI assistance for BAA, the ICCs for both methods among all reviewers exceeded 0.99, and the mean differences were close to zero (Figs. 5 and 6).

## Discussion

Bone age serves as a quantitative measure of skeletal development maturity.^1^ The utilization of X-ray wrist images for BAA in children become widespread.^10–14^ In the past, manual BAA methods required observers to meticulously compare or score individual bones.^16^ DL offers a faster and more consistent solution. We categorized multiple observers into groups, scrutinized and compared their diagnostic accuracy both with and without AI assistance, and assessed inter-observer consistency and intra-observer reproducibility. The findings reveal that experienced radiologists can enhance the precision of BAA with the aid of AI. Simultaneously, AI can mitigate inter-observer variability and enhance intra-observer reproducibility.

AI technology stands as a prominent application within the realm of medical imaging, including the diagnosis of lung nodules and the detection of bladder cancer.^17,18^ DL can precisely quantify the shape and position of each target bone in the wrist for BAA, with its development dating back to 2017.^19^ Presently, researchers construct algorithmic models to predict BA rapidly and accurately.^20^ drawing from a vast repository of images.^9,21^ Spampinato et al.^9^ were pioneers in exploring the application of DL to medical images, and they demonstrated an average deviation of about 0.8 years when compared to manual evaluations. In 2020, Reddy et al.^22^ employed a publicly provided anonymized dataset from the Radiological Society of North America pediatric bone age challenge.^2^ The MAEs between the models for the whole hand and index finger were comparable (0.392 years vs. 0.425 years, *p* = 0.14). Both BA values were significantly smaller than those obtained by three pediatric radiologists from single-finger radiographs (0.667 years, *p* < 0.0001). Larson et al.^21^ developed a DL model for BAA based on a comparison with 12,611 clinical hand radiographs using the Greulich and Pyle (GP) atlas and corresponding clinical radiology reports. The mean difference between the model’s BAA radiographs and reviewers was 0 years, with a mean RMSE and MAE of 0.63 and 0.50 years, respectively. All assessments fell within the 95% limits of agreement with each other. The Residual Network model effectively extracts X-ray bone image features and autonomously determines bone age, boasting an impressive BA prediction accuracy of 97.6% and a MAE of 0.455 year.^12^ AI models have consistently demonstrated high accuracy in BAA,^21–23^ and this study’s results reaffirm this fact. Radiologists can enhance their diagnostic accuracy in BA evaluation with the assistance of AI models.

Environmental and ethnic factors exert varying degrees of influence on bone development, leading to differing outcomes in BAA. We employed two distinct BAA methods, primarily suited for Chinese children. Both the TW3 method and the RUS-CHN method are widely utilized for the assessment of preschool children. The TW3 method evaluates and scores the maturity of each region of interest bone and drew reference data from children residing in Europe and America, with publication occurring in 2001.^4^ The TW3 method is a quantitative approach that scores and sums 20 hand-wrist bones, which characterized by strong objectivity, resulting in highly accurate assessments with a precision of less than one month.^24^ However, it is time-consuming and entails a complex evaluation process. Several studies have affirmed the high accuracy of the TW3 method for BAA has high accuracy.^3,25^ In a British children’s sample, CA was underestimated in females beyond the age of 3 years, resulting in significant differences between BA and CA (−0.43 years, *p* < 0.001), while no such differences were observed in males (0.01 years, *p* = 0.760).^3^ Based on an analysis of 9059 clinical left hand radiographs, an optimized TW3-AI system for BAA exhibited strong concordance with the overall assessment of reviewers, with a RMSE of 0.50 years.^25^ In our study, with the aid of the AI model system, the RMSE of observations by mid-level doctors decreased from 0.358 to 0.151. This further underscores that AI has the potential to narrow the disparity in BAA results compared to the reference standard in the TW3 method, thereby assisting physicians in enhancing diagnostic accuracy. In 2006, researchers^5^ revised the standards based on the TW3 method and established the RUS-CHN method. using samples from urban areas in China Building on the original bone development framework of the TW3 method, the RUS-CHN method identifies new maturity characteristics, which better align with the actual skeletal conditions of children during their rapid growth and development. It also subdivides the long-term fusion process of the radius and ulna into five distinct grades, thereby enhancing accuracy throughout the entire growth and development period.^26^ The RUS-CHN method, necessitates more steps, consumes additional time during the evaluation process, and is challenging to master. In a preliminary study conducted by our team involving 390 preschool children, it was observed that while the TW3 method outperformed the RUS-CHN method, it was not entirely reliable on its own. This is because both methods tended to overestimate the age of both sexes. Nevertheless, the median difference of the TW3 method approached zero.^27^ In the current study, when observers used the RUS-CHN method, both with and without AI assistance, the RMSE was 0.359 and 0.148, while the MAE was 0.309 and 0.113, respectively, signifying a high level of diagnostic performance. Moreover, with the aid of AI, observer diagnostic accuracy can be further enhanced.

Applying AI systems to BAA presents two primary challenges, namely ensuring consistency in both inter- and intra-observer evaluations. in an investigation involving American children, researchers compared the BAA performance of a group of pediatric radiologists with and without AI support. With AI assistance, BAA accuracy improved, with an overall accuracy of 68.2% compared to 63.6%, and an accuracy of 98.6% within 1 year compared to 97.4%. Additionally, the ICC with AI was 0.9951, whereas without AI, it was 0.9914.^10^ Lee KC et al.^28^ discovered that a deep learning-based model exhibited accuracy in BAA for a total of 102 hand radiographs. Furthermore, it appeared to enhance clinical efficacy by improving inter-observer reliability, which slightly increased the ICC of the two observers from 0.945 to 0.990 with AI. More recently, Wang X et al.^15^ concluded that an AI model enhances both the accuracy and consistency of BAA for physicians of all experience levels. The accuracies of senior, mid-level, and junior physicians were significantly better with AI assistance than without AI assistance (MAEs of 0.325, 0.344, and 0.370 vs. 0.403, 0.469, and 0.755, respectively). Moreover, their consistency results were significantly higher with AI assistance than without AI assistance (ICCs of 0.996, 0.996, and 0.992 vs. 0.987, 0.989, and 0.941, respectively). In this study, for the inter-observer agreement comparison, with the aid of AI, the ICC values for both BAA methods reached 0.991 in the 1st interpretation. Regarding intra-observer reproducibility between the 1st and 2nd interpretation, the ICC results were elevated to 0.998 for the TW3 method and up to 0.997 for the RUS-CHN method (Reviewer 4). And the Bland-Altman plots showed an excellent agreement among the reviewers in both two methods. The Utilizing AI-assisted software in BAA can help reviewers mitigate both inter-observer variability and intra-observer variability.

The development of AI software has simplified and expedited the BAA process. Numerous studies have compared BAA differences between AI tools and radiologists.^13,16,21,28–30^ Their findings confirm that AI can enhance diagnostic accuracy. However, relying solely on AI results without confirmation from a radiologist is not considered reliable.^31^ In such cases, AI software is designed to assist radiologists in making faster and more accurate diagnoses rather than replacing radiologists outright. two scenarios were established for observers, one with and one without the AI model system, and BAA accuracy was calculated separately. Our results align with previous findings and further substantiate that AI can help radiologists enhance the accuracy of BAA, particularly in preschool children, using both the TW3 and RUS-CHN methods.

The present study has several limitations: 1) This is a single-center, cross-sectional study with a small sample size, focused only on a specific population aged 3–6 years in China. 2) The study exclusively compared the TW3 and RUS-CHN methods, but other methods like the GP method, which is commonly used in various regions and hospitals, were not considered. 3) The observers in this study were mid-level attending physicians, and there was no comparison with physicians of other levels, such as junior and senior physicians. 4) The timing of bone age assessment was not documented, even though previous studies have found that AI can reduce assessment time. Comparative time consumption should be considered. Therefore, more in-depth multicenter studies are necessary to validate these findings, incorporating various BAA methods and observers with different levels of experience in future research.

During the process of BAA for preschool children, the use of AI model systems can significantly improve not only the diagnostic accuracy of physicians but also the consistency among observers and the reproducibility within observers. As a result, AI model systems hold great promise for X-ray hand-wrist bone age assessment and are a valuable tool in the clinical work of radiologists.

## Data Availability

The raw data supporting the conclusions of this article will be made available by the authors without undue reservation.
